# Whole-genome sequencing of clinical methicillin-resistant Staphylococcus aureus isolates across hospitals in Mwanza, Tanzania

**DOI:** 10.1099/acmi.0.001127.v5

**Published:** 2026-06-12

**Authors:** Vitus Silago, Lawrence Mapunda, Prisca Damiano, Katarina Oravcova, Louise Matthews, Benson R. Kidenya, Stephen E. Mshana, Jeremiah Seni, Heike Claus

**Affiliations:** 1 Department of Microbiology and Immunology, Weill Bugando School of Medicine, Catholic University of Health and Allied Sciences, P. O. Box 1464, Mwanza, Tanzania; 2 Institute for Hygiene and Microbiology, University of Würzburg, Sanderring 2, 97070 Würzburg, Germany; 3 Department of Molecular Virology, National Public Health Laboratory, P. O. Box 9083, Dar es Salaam, Tanzania; 4 School of Biodiversity, One Health and Veterinary Medicine, University of Glasgow, Glasgow, UK; 5 Department of Biochemistry and Molecular Biology, Weill Bugando School of Medicine, Catholic University of Health and Allied Sciences, P. O. Box 1464, Mwanza, Tanzania

**Keywords:** antimicrobial resistance genes, methicillin-resistant* Staphylococcus aureus*, National Action Plan on Antimicrobial Resistance, virulence factors, whole-genome sequencing

## Abstract

Methicillin-resistant *Staphylococcus aureus* (MRSA) is a leading cause of both healthcare-associated and community-acquired infections, characterized by limited treatment options and substantial morbidity and mortality, particularly in low- and middle-income countries such as Tanzania. However, genomic data, which provides valuable insights into the pathogen’s genetic landscape, remains scarce. This study utilized whole-genome sequencing (WGS) to examine local molecular epidemiological profiles of 14 MRSA isolates isolated from blood, urine and pus samples during (*n*=6; June 2019–June 2020) and after (*n*=8; March–August 2023) the implementation of the National Action Plan on Antimicrobial Resistance in Mwanza, Tanzania. All isolates belonged to sequence type 8 (ST8), and 92.9% (13/14) were spa type *t*1476. Phenotypic and genotypic antimicrobial resistance results were consistent for all antibiotics tested with the exception of tetracycline. Pairwise SNP analysis revealed genetic diversity, with SNP differences ranging from 0 to 99. Maximum likelihood phylogeny analysis based on SNP data identified two closely related MRSA pairs, one from multiple wards while the other confined within the same ward in the same hospital. In conclusion, WGS revealed that all MRSA isolates belonged to ST8 with substantial genetic diversity and phylogenetic analysis revealed two closely related MRSA pairs, suggesting a transmission event, acquisition from a shared source or circulation of a closely related lineage. These findings highlight the importance of genomic surveillance for uncovering MRSA transmission patterns and guiding infection prevention efforts.

**Impact Statement**

This study advances methicillin-resistant *Staphylococcus aureus* (MRSA) research in Tanzania by providing a comprehensive genomic assessment of MRSA isolates linked to the implementation of the National Action Plan on Antimicrobial Resistance. By uncovering a predominance of MRSA ST8 spa type *t*1476 lineage with evidence of closely related MRSA pairs, the analysis highlights the presence of gaps in infection prevention and control practices. These findings demonstrate the practical value of integrating whole-genome sequencing into routine antimicrobial resistance (AMR) surveillance and underscore its potential to guide targeted interventions and strengthen future national AMR response efforts.

## Data Summary

The whole-genome sequences generated in this study are available under BioProject ID: PRJNA1241466 (BioSamples: SAMN47557156 – 69) in the NCBI GenBank.

## Introduction

Antimicrobial resistance (AMR) has emerged as one of the most significant threats to global public health [1], with methicillin-resistant *Staphylococcus aureus* (MRSA) being a particularly formidable challenge [2]. MRSA is a leading cause of both hospital- and community-acquired infections and is associated with severe morbidity, mortality and a substantial economic burden on healthcare systems worldwide [3]. Its ability to persist and adapt to various environments, to evade immune defenses and to develop resistance to multiple antibiotics has made it a focal point in the global fight against AMR [2,4]. Sequence type 8 (ST8) is of particular concern, as it has been described to disseminate widely locally [5] and globally [6,8] and to exhibit remarkable multidrug resistance and adaptability, making treatment more challenging and contributing to its continued spread [9]. The spread and the burden of MRSA is significant in resource-constrained regions like Africa, where healthcare diagnostic infrastructures and surveillance systems are still evolving [10].

In Africa, including Tanzania, the rise of MRSA infections highlights the need for targeted interventions, rigorous monitoring and enhanced laboratory capabilities to tackle this growing threat. In response to the escalating AMR crisis, Tanzania implemented its first National Action Plan on Antimicrobial Resistance (NAP-AMR) from 2017 to 2022 [11], aligning its objectives with the World Health Organization’s Global Action Plan on AMR [12]. This ambitious initiative aimed to strengthen AMR surveillance, promote rational antibiotic use and bolster infection prevention and control (IPC) measures across healthcare settings, notably zonal and regional referral hospitals [11]. Additionally, in collaboration with the Supporting NAP-AMR (SNAP-AMR) project, we extended AMR surveillance through culture and antimicrobial susceptibility testing (AST) services to district hospitals in Mwanza region. Mwanza, a key region in Northwestern Tanzania, has become a focal area in these efforts due to its high prevalence of bacterial infections and AMR [13,14], including MRSA, to mitigate its risks to individual patient outcomes and the broader public health landscape [15].

Previous whole-genome sequencing (WGS) studies of MRSA isolates from various Tanzanian regions, excluding Mwanza, have consistently identified MRSA ST8 spa type *t*1476 as the predominant strain [16]. This strain has been recovered from the nasopharynx/anterior nares of newly diagnosed HIV-positive individuals (April 2017–May 2018) [16], from diverse clinical specimens (January 2020–December 2021) [5,17] and from chronic leg ulcer infections (August 2022–April 2023) [18]. However, despite ongoing AMR mitigation efforts by NAP-AMR and the genomic data generated by these previous Tanzanian studies, a critical knowledge gap persists. Specifically, comprehensive data from the Mwanza region remains scarce. Therefore, this study aimed to acquire molecular epidemiological characteristics of MRSA isolates isolated from patients with bloodstream infections (BSIs), urinary tract infections (UTIs) and skin and soft tissue infections (SSTIs) during and after NAP-AMR from five healthcare facilities in Mwanza, Tanzania.

## Methods

### Study design, duration, population and setting

A cross-sectional hospital-based study was conducted among patients with clinical symptoms of BSIs, UTIs and SSTIs attending or admitted at a zonal referral hospital, a regional referral hospital and three district hospitals in Mwanza, Tanzania from June 2019 to June 2020 (during NAP-AMR) and from March to August 2023 (after NAP-AMR) [14,19]. The zonal and regional referral hospitals were referred to as ‘higher-tier hospitals’, whereas district hospitals were referred to as ‘lower-tier hospitals’.

Briefly, at each study site, conventional cultures of blood, urine and pus were performed, followed by biochemical identification and AST using a standardized protocol. These procedures enabled the isolation of bacterial pathogens, species-level identification and determination of susceptibility patterns for BSIs, UTIs and SSTIs. Results from culture and AST were promptly communicated to support clinical management. For *S. aureus* isolates, AST included cefoxitin testing to identify MRSA isolates.

All bacterial isolates were collected from the sentinel hospitals to the Catholic University of Health and Allied Sciences (CUHAS) Microbiology Laboratory, where they were stored at −80 °C before duplicate sets were shipped to the Institute for Hygiene and Microbiology at the University of Würzburg, Germany, for further analysis. In Würzburg, isolates, including *S. aureus*, were revived by subculture on 5% sheep blood agar at 35±2 °C for 24 h. They were subsequently re-identified using Vitek MS (bioMérieux, Nürtingen, Germany) and re-tested for antimicrobial susceptibility using the Vitek 2 system (bioMérieux, Nürtingen, Germany). For *S. aureus*, AST-P654 cards containing oxacillin were used for AST and MRSA detection. Minimum inhibitory concentrations were interpreted according to the European Committee on Antimicrobial Susceptibility Testing guidelines [20]. In total, 45 *S*. *aureus* were isolated, 22 during and 23 after NAP-AMR, of which 14 were MRSA. All MRSA isolates were included in this study for WGS.

### Whole genome sequencing, phylogenetic and annotation analyses

DNA extraction using Wizard^®^ Genomic DNA Purification Kit (Promega, Walldorf, Germany) of all MRSA was performed as instructed by the manufacturer. DNA samples with A260/A280 and A260/A230 ratios ≥1.80 measured by Nanodrop Spectrophotometer (Thermo Fisher Scientific, Darmstadt, Germany) were stored at −20 °C until WGS. Sequencing libraries were generated with the Illumina Nextera XT DNA library preparation kit. The WGS was performed on an Illumina NextSeq 550 instrument (Illumina, San Diego, CA, USA) using a 2×150 bp paired-end kit. With Ridom SeqSphere+v0.8.4.0 (Ridom GmbH, Münster, Germany; https://www.ridom.de/seqsphere/ug/v40/Overview.html) [21], raw sequences were quality checked using FastQC v0.11.9 [22] and trimmed (removal of adapters and poor-quality sequences) using Trimmomatic v0.39 [23] and subjected to *de novo* assembly with Velvet v1.1.04 [24]. The sequenced MRSA isolates had an average genome size of 2.86 Mbp (range: 2.75–2.95 Mbp), with a mean 32.69 mol% G+C content (range: 32.6–32.9 mol%). The average N50 was 48.43 Kbp (range: 26–77 Kbp), and the mean number of contigs was 140.14 (range: 86–217). The *S. aureus* PubMLST database (https://pubmlst.org/organisms/staphylococcus-aureus) was used to identify the MLST ST [25], and spa types were analysed with StaphType [26] within Ridom SeqSphere+.

Sequence annotation was conducted using Prokka [27], and the resulting annotated genomes were generated as genetic feature files (GFF). These GFF files were used as input for Panaroo v1.6.0 [28], where a core genome alignment was generated using the MAFFT aligner [29] with default parameters in strict mode. This alignment was subsequently used as input for Gubbins v3.4.1 [30] to detect and mask recombination. The recombination-filtered alignment from Gubbins was then processed with SNP sites to extract SNP positions, and the resulting SNP-only alignment was used as input to generate a maximum likelihood phylogenetic tree with IQ-TREE v3.1.1 [31] using the GTR+ASC model according to Bayesian information criteria (BIC). ModelFinder [32] was used to identify the best-fitting evolutionary model based on the BIC, and bootstrap support values were calculated from 1,000 replicates. The final tree was visualized with Interactive Tree of Life (iTOL) v7 [33] and rooted at the midpoint. With ≤10 SNPs set as clustering threshold. Additionally, to examine if the same MRSA ST8 lineage circulates in Tanzania, we performed genomic comparison of MRSA ST8 isolates from our study and MRSA ST8 isolates reported in previous studies by Manyahi *et al*. (22 genomes) [16], Geofrey *et al*. (66 genomes; BioProject ID: PRJEB71932) [17] and Omar *et al*. (4 genomes; BioProject ID: PRJEB75012) [18].

The PlasmidFinder 2.1 [34,35] was used for screening of plasmid replicons which were further reconstructed and typed using MOB-suite v3.1.9 [36]. Plasmid replicons with a Mash neighbour distance of <0.01 [37] were recorded and included for downstream analysis of AMR and virulence factor (VF) genes carriage. Annotation of plasmid replicons harbouring AMR genes (ARGs) was conducted using the Comprehensive Antibiotic Resistance Database (CARD) [38], accessed via Proksee (https://proksee.ca/) [39]. VirulenceFinder 2.0 [34] and ResFinder 4.6.0 [34,40] of the Center for Genomic Epidemiology (CGE: accessed between 2 November 2024 and February 10, 2025) were used for the detection of genes coding for VFs and AMR, respectively. Additionally, the Resistance Gene Identifier [38] from the CARD was used to identify AMR genes that were not detected by ResFinder 4.6.0. This analysis was guided by phenotypic resistance patterns, and only ARGs with perfect or strict hits (≥95% identity) were recorded [38]. A pangenome analysis was performed by using a web-based tool, Integrated Prokaryotes Genome and pan-genome Analysis (IPGA) v1.09 [41] available at the National Microbiology Data Center (Beijing, China; https://nmdc.cn/ipga/) using genome assembled to identify core and accessory genes and understand evolutionary relationships of MRSA isolates.

### Statistical analysis

Data were first recorded in Microsoft Excel for cleaning and coding before being transferred to STATA 15.0 (Stata Corp LLC, College Station, Texas, USA) for analysis. The descriptive analysis of the categorical variables entailed summarizing the data through frequency distributions, proportions and percentages. Proportions were statistically compared using the Chi-square test.

### Ethical considerations

The data utilized in this study were derived from prior research projects that received ethical approval from the joint CUHAS and Bugando Medical Centre (BMC) Research Ethics and Review Committee (CREC), under the approval number: CREC/318/2018 (during NAP-AMR) and CREC/654/2023 (after NAP-AMR). Additionally, this study was approved by the National Health Research Ethics Committee (NatHREC) with approval numbers NIMR/HQ/R.8a/Vol.IX/3017 (during NAP-AMR) and NIMR/HQ/R.8a/Vol.IX/4613 (after NAP-AMR). Originally, all participants were enrolled following the provision of voluntarily informed written consent forms. Furthermore, culture and AST results were promptly shared with healthcare providers to facilitate timely and effective patient management, aligning with the study’s commitment to both scientific rigour and ethical responsibility.

## Results

### Brief descriptions of patients with MRSA infections

A total of 14 MRSA isolates were isolated from clinical samples during (*n*=6) and after (*n*=8) the implementation of NAP-AMR. These infections included SSTIs (*n*=6), UTIs (*n*=5) and BSIs (*n*=3). The majority of patients who developed MRSA infections were treated at a higher-tier hospital (*n*=9) and most patients were outpatients (*n*=8). Ten patients were on antibiotics at the time of sampling of which eight patients had a history of antibiotic use within the past 3 months (Table 1). The most commonly recorded antibiotics at the time of sampling were ceftriaxone (*n*=3), ciprofloxacin (*n*=2) and ampicillin/cloxacillin (ampiclox; *n*=2).

**Table 1.** Patients with MRSA infections during and after NAP-AMR

Isolate ID	Source	Patient category	History of antibiotic	Current on antibiotic	Isolation date	Study period	Facility name	Facility level
**TU016**	Urine	Outpatient	Yes	Yes	15 September 2019	During NAP-AMR	SDDH	Lower-tier
**TU020**	Urine	Outpatient	No	Yes	26 September 2019	During NAP-AMR	SDDH	Lower-tier
**TU135**	Urine	Outpatient	Yes	Yes	14 October 2019	During NAP-AMR	BMC	Higher-tier
**TB032**	Blood	Inpatient	Yes	Yes	20 October 2019	During NAP-AMR	BMC	Higher-tier
**TP018**	Pus	Outpatient	No	No	24 January 2020	During NAP-AMR	MisDH	Lower-tier
**TU075**	Urine	Outpatient	No	Yes	29 March 2020	During NAP-AMR	MisDH	Lower-tier
**TP006B**	Pus	Outpatient	No	No	18 April 2023	After NAP-AMR	BMC	Higher-tier
**TP044B**	Pus	Outpatient	No	No	19 May 2023	After NAP-AMR	BMC	Higher-tier
**TP062B**	Pus	Inpatient	Yes	Yes	14 June 2023	After NAP-AMR	BMC	Higher-tier
**TP058B**	Pus	Inpatient	Yes	Yes	15 June 2023	After NAP-AMR	BMC	Higher-tier
**TP069B**	Pus	Inpatient	Yes	Yes	16 June 2023	After NAP-AMR	BMC	Higher-tier
**TB045B**	Blood	Inpatient	Yes	Yes	16 June 2023	After NAP-AMR	BMC	Higher-tier
**TB081B**	Blood	Inpatient	Yes	Yes	28 June 2023	After NAP-AMR	BMC	Higher-tier
**TU141B**	Urine	Outpatient	No	No	11 July 2023	After NAP-AMR	MDH	Lower-tier

The isolates are arranged by date of isolation. The isolate IDs comprise a ‘T’ denoting the country of origin (Tanzania), a letter indicating the specimen type: U (urine), B (blood) and P (pus), and a numeric identifier. A ‘B’ suffix indicates sample collection after NAP-AMR.

MDH, Magu District Hospital; MisDH, Misungwi District Hospital; SDDH, Sumve designated District Hospital.

### Genetic typing of MRSA isolated from the clinical samples during and after NAP-AMR

All sequenced MRSA isolates (*n*=14) belonged to ST8, with the majority classified as spa type *t*1476 (92.9%, 13/14). One MRSA strain (TP69B) isolated from a pus sample after NAP-AMR was spa type *t*11148. Plasmid replicons were detected in all isolates, yielding a total of 66 plasmid replicons. The most prevalent replicon was rep7c (21.2%, 14/66), followed by rep10 (18.2%, 12/66), rep5a (15.1%, 10/66) and rep16 (15.1%, 10/66) (Table 2).

**Table 2.** Sequence types, spa types and plasmid replicons of MRSA isolates isolated from clinical samples

Variable	Category	Frequency (n)	Percentage (%)
**Sequence type (ST**)	ST-8	14	100
**Spa type**	*t*1476	13	92.9
	*t*11148	1	7.1
**Plasmid replicons (*n*=66**)	rep7c	14	21.2
	rep10	12	18.2
	rep5a	10	15.1
	rep16	10	15.1
	rep15	9	13.6
	rep19	6	9.1
	rep7a	5	7.6

STs were assigned using the *Staphylococcus aureus* PubMLST database, based on allelic profiles of seven housekeeping genes. Spa types were determined by analysis of the polymorphic X region of the *S. aureus* spa gene using StaphType within Ridom SeqSphere+. Plasmid replicons were identified using PlasmidFinder 2.1.

**Fig. 1.**
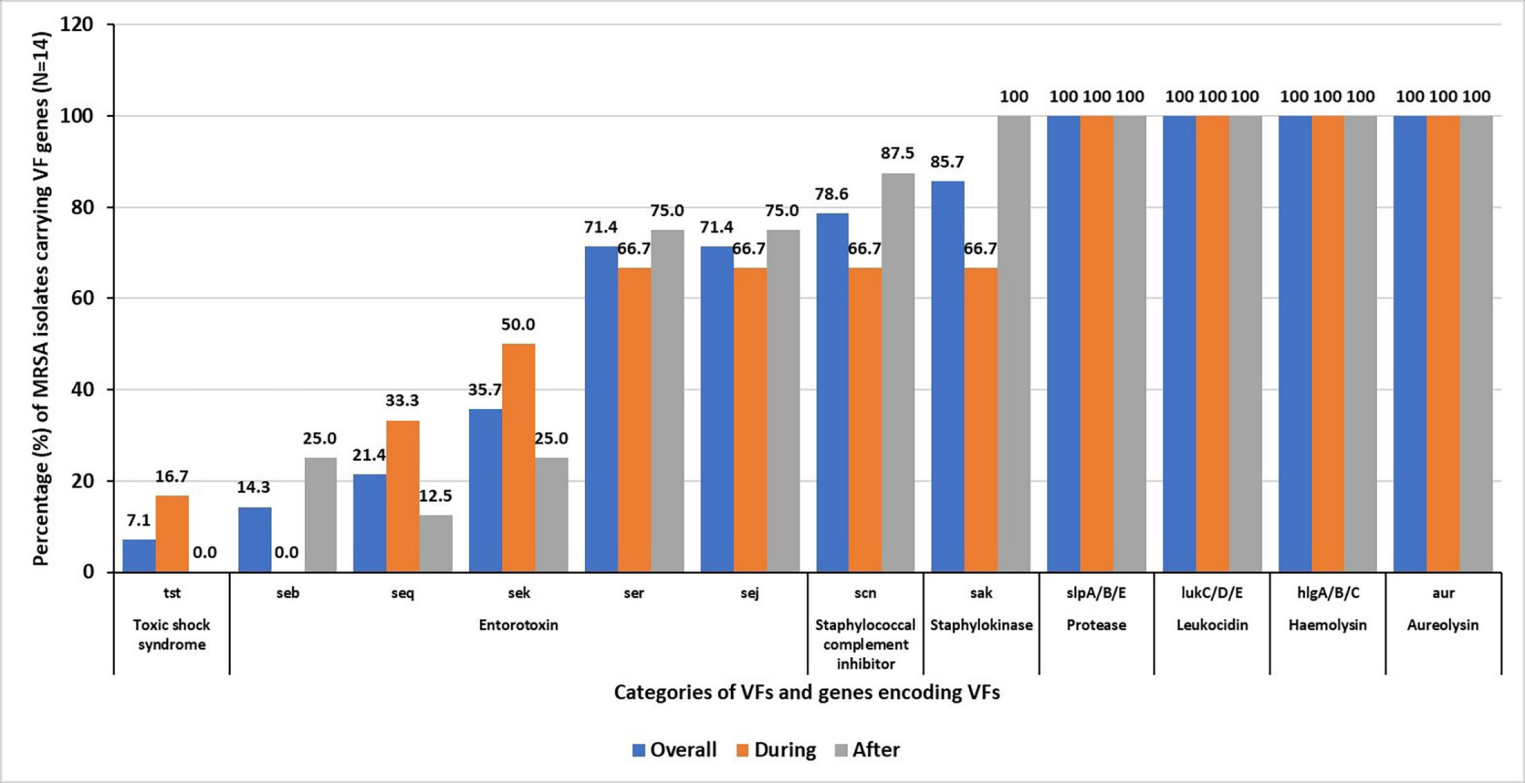
Distribution of VF-encoding genes among MRSA clinical isolates collected during and after NAP-AMR implementation in Mwanza, Tanzania. Blue, orange and grey bars indicate the percentage of MRSA isolates carrying each VF gene across the overall cohort (*N*=14), during NAP-AMR (*n*=6) and after NAP-AMR (*n*=8), respectively. Genes are organized by functional category: toxic shock syndrome (*tst*), enterotoxins (*seb*, *seq*, *sek*, *ser* and *sej*), staphylococcal complement inhibitor (*scn*), staphylokinase (*sak*), protease (*slp*A/B/E), leukocidin (*luk*C/D/E), haemolysin (*hlg*A/B/C) and aureolysin (*aur*).

All MRSA isolates were examined for carriage of genes encoding VFs. A total of 110 VF genes, classified into eight functional categories, were identified. A higher proportion of VF genes was detected among MRSA isolates recovered after NAP-AMR (58.2% [*n*=64] vs. 41.8% [*n*=46], *P*=0.086). Generally, the *aur*, *hlg*A/B/C, *luk*C/D/E and *spl*A/B/E genes encoding aureolysin, hemolysin, leukocidin and protease, respectively, were detected in all isolates. Moreover, the genes *sak*, *scn*, *sej* and *ser*, encoding staphylokinase, staphylococcal complement inhibitors and certain enterotoxins, were present in 85.7% (*n*=12), 78.6% (*n*=11), 71.4% (*n*=10) and 71.4% (*n*=10) isolates, respectively. Interestingly, only one strain (TU75), isolated from a urine sample during NAP-AMR, harboured the *tst* gene encoding the toxic shock syndrome toxin-1 (TSST-1) (Fig. 1).

### Antimicrobial resistance genes in MRSA clinical strains

**Fig. 2.**
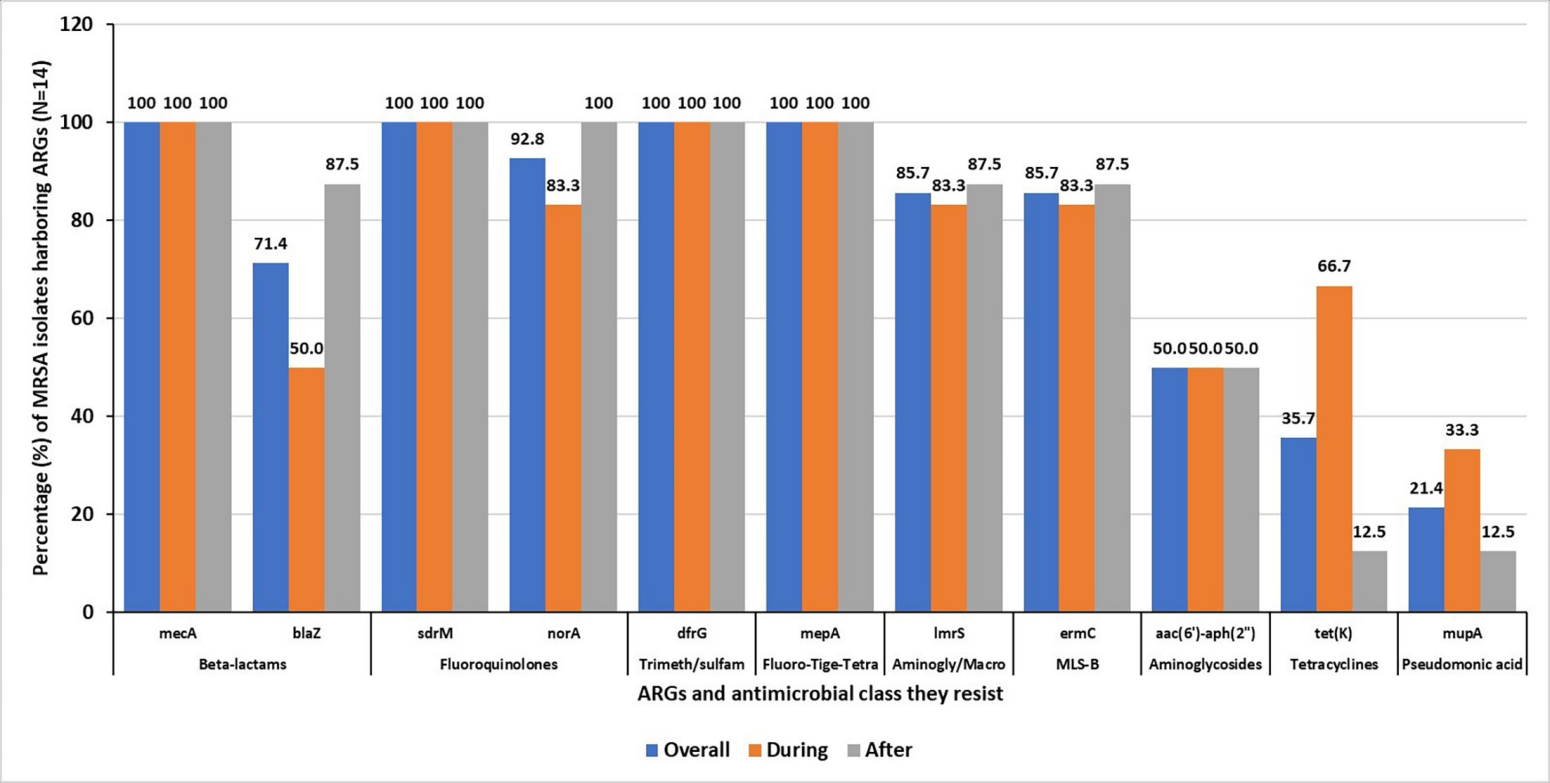
Distribution of ARGs among MRSA clinical isolates collected during and after NAP-AMR implementation in Mwanza, Tanzania. Blue, orange and grey bars indicate the percentage of MRSA isolates carrying each ARG across the overall cohort (*N*=14), during NAP-AMR (*n*=6) and after NAP-AMR (*n*=8), respectively. Genes are organized by antimicrobial class they resist: β-lactams (*mec*A, *bla*Z), fluoroquinolones (*sdr*M, *nor*A), trimethoprim/sulfamethoxazole (*dfr*G), fluoroquinolone-tigecycline-tetracycline (*mep*A), aminoglycoside/macrolide (*lmr*S), macrolide-lincosamide-streptogramin B (MLS-B; *erm*C), aminoglycosides (*aac*(6′)-aph(2″)), tetracyclines (*tet*(K)) and pseudomonic acid (*mup*A).

A total of 118 ARGs were identified across all MRSA isolates, with a higher proportion detected in isolates recovered after NAP-AMR [56.8% (*n*=66) vs. 43.2% (*n*=51), *P*=0.141]. The *mec*A, *sdr*M, *dfr*G and *mep*A genes, which confer resistance to β-lactams, fluoroquinolones, trimethoprim-sulfamethoxazole and a combination of fluoroquinolones, tigecycline and tetracycline (Fluoro-Tige-Tetra), respectively, were detected in all isolates. Additionally, the fluoroquinolone resistance gene *nor*A was found in 92.8% (13/14) of the isolates. The *lmr*S gene, associated with aminoglycoside/macrolide resistance, and the *erm*(C) gene, linked to resistance against macrolides, lincosamides and streptogramin B (MLS-B), were both identified in 85.7% (12/14) of the isolates. The *bla*Z gene, which mediates β-lactam resistance, was present in 71.4% (10/14). Furthermore, the *aac*(6′)-aph(2′), *tet*(K) and *mup*A genes, conferring resistance to aminoglycosides, tetracyclines and pseudomonic acid (mupirocin), respectively, were detected in 50.0% (7/14), 35.7% (5/14) and 21.4% (3/14) of isolates (Fig. 2).

### Correlation between phenotypic and genotypic antimicrobial resistance, and antimicrobial resistance patterns

All MRSA isolates exhibiting phenotypic resistance to trimethoprim-sulfamethoxazole and levofloxacin harboured the *dfr*G and *sdr*M genes, respectively. Additionally, high resistance rates were observed towards erythromycin (85.7%, 12/14) by *erm*(C) and gentamicin (92.8%, 13/14) by *aac*(6'′)-aph(2′) and/or *lmr*S, while mupirocin resistance was detected in 21.4% (3/14). Moreover, phenotypic AMR patterns correlated with genotypic resistance predictions, except for tetracycline. Resistance to tetracycline was noted in 42.8% (6/14) of isolates of which five harboured *tet*(K) and *mep*A, while one isolate carried only *mep*A, which encodes an efflux mechanism for Fluoro-Tige-Tetra. Of note, six MRSA isolates harboured mepA but were tetracycline susceptible. Inducible clindamycin resistance (iCLI-R) encoded by *erm*(C) gene was identified in 85.7% (12/14) of the isolates. All MRSA isolates were resistant to at least three antimicrobial agents, and the most prevalent resistance pattern was ERY-GEN-LVX-SXT (*n*=6). Another six resistance patterns were identified in only one (TU016, TU020, TB045-B and TP006-B) or two (TU135, TB032, TP044-B and TP062-B) MRSA isolates (Table 3). Neither phenotypic nor genotypic predicted resistance was observed against daptomycin, fosfomycin, fusidic acid, linezolid, teicoplanin, tigecycline and vancomycin.

**Table 3.** Correlation between phenotypic susceptibility and presence of ARGs, inducible clindamycin resistance and resistance patterns of MRSA isolates

Isolates	Erythromycin		Gentamicin		Levofloxacin		Mupirocin		Trimethoprim-sulfamethoxazole		Tetracycline		iCLI-R	Resistance patterns
	PR	ARGs	PR	ARGs	PR	ARG	PR	ARGs	PR	ARGs	PR	ARGs		
TU016	S	NF	R	*lmr*S	R	*mep*A, *nor*A, *sdr*M	S	NF	R	*dfr*G	R	*mep*A*, tet*(K)	Neg	GEN-LVX-SXT-TCY
TU020	R	*erm*(C)	S	NF	R	*mep*A, *nor*A, *sdr*M	S	NF	R	*dfr*G	R	*mep*A*, tet*(K)	Pos	ERY-LVX-SXT-TCY
TU135	R	*erm*(C)	R	*aac*(6')-aph(2’), *lmr*S	R	*mep*A, *nor*A, *sdr*M	R	*mup*A	R	*dfr*G	R	*mep*A*, tet*(K)	Pos	ERY-GEN-LVX-MUP-SXT-TCY
TB032	R	*erm*(C)	R	*aac*(6')-aph(2’), *lmr*S	R	*mep*A, *sdr*M	R	*mup*A	R	*dfr*G	R	*mep*A*, tet*(K)	Pos	ERY-GEN-LVX-MUP-SXT-TCY
TP018	R	*erm*(C)	R	*aac*(6')-aph(2’), *lmr*S	R	*mep*A, *nor*A, *sdr*M	S	NF	R	*dfr*G	**S**	***mep*A**	Pos	ERY-GEN-LVX-SXT
TU075	R	*erm*(C)	R	*lmr*S	R	*mep*A, *nor*A, *sdr*M	S	NF	R	*dfr*G	**S**	***mep*A**	Pos	ERY-GEN-LVX-SXT
TP006-B	S	NF	R	*lmr*S	R	*mep*A, *nor*A, *sdr*M	S	NF	R	*dfr*G	**S**	***mep*A**	Neg	GEN-LVX-SXT
TP044-B	R	*erm*(C)	R	*aac*(6')-aph(2’), *lmr*S	R	*mep*A, *nor*A, *sdr*M	S	NF	R	*dfr*G	R	*mep*A	Pos	ERY-GEN-LVX-SXT-TCY
TP062-B	R	*erm*(C)	R	*lmr*S	R	*mep*A, *nor*A, *sdr*M	S	NF	R	*dfr*G	R	*mep*A*, tet*(K)	Pos	ERY-GEN-LVX-SXT-TCY
TP058-B	R	*erm*(C)	R	*aac*(6')-aph(2’), *lmr*S	R	*mep*A, *nor*A, *sdr*M	S	NF	R	*dfr*G	**S**	***mep*A**	Pos	ERY-GEN-LVX-SXT
TP069-B	R	*erm*(C)	R	*aac*(6')-aph(2’), *lmr*S	R	*mep*A, *nor*A, *sdr*M	S	NF	R	*dfr*G	**S**	***mep*A**	Pos	ERY-GEN-LVX-SXT
TB045-B	R	*erm*(C)	R	*lmr*S	R	*mep*A, *nor*A, *sdr*M	R	*mup*A	R	*dfr*G	**S**	***mep*A**	Pos	ERY-LVX-MUP-SXT
TB081-B	R	*erm*(C)	R	*aac*(6')-aph(2’), *lmr*S	R	*mep*A, *nor*A, *sdr*M	S	NF	R	*dfr*G	S	*mep*A	Pos	ERY-GEN-LVX-SXT
TU141-B	R	*erm*(C)	R	NF	R	*mep*A, *nor*A, *sdr*M	S	NF	R	*dfr*G	S	*mep*A	Pos	ERY-GEN-LVX-SXT
**% R**	**85.7**	**na**	**92.8**	**na**	**100**	**na**	**21.4**	**na**	**100**	**na**	**42.7**	**na**	**85.7**	**na**

The isolate IDs comprise a ‘T’ denoting the country of origin (Tanzania), a letter indicating the specimen type: U (urine), B (blood) and P (pus) and a numeric identifier. A ‘B’ suffix indicates sample collection after NAP-AMR.

ERY, erythromycin; GEN, gentamicin; LVX, levofloxacin; MUP, mupirocin; NA, not applicable; Neg, negative; NF, not found; Pos, positive; PR, phenotypic resistance; %R, percentage resistance; R, resistant; S, sensitive; SXT, trimethoprim-sulfamethoxazole; TCY, tetracycline.

### Carriage of AMR and VF genes within rep-plasmids among sequenced MRSA isolates

**Fig. 3.**
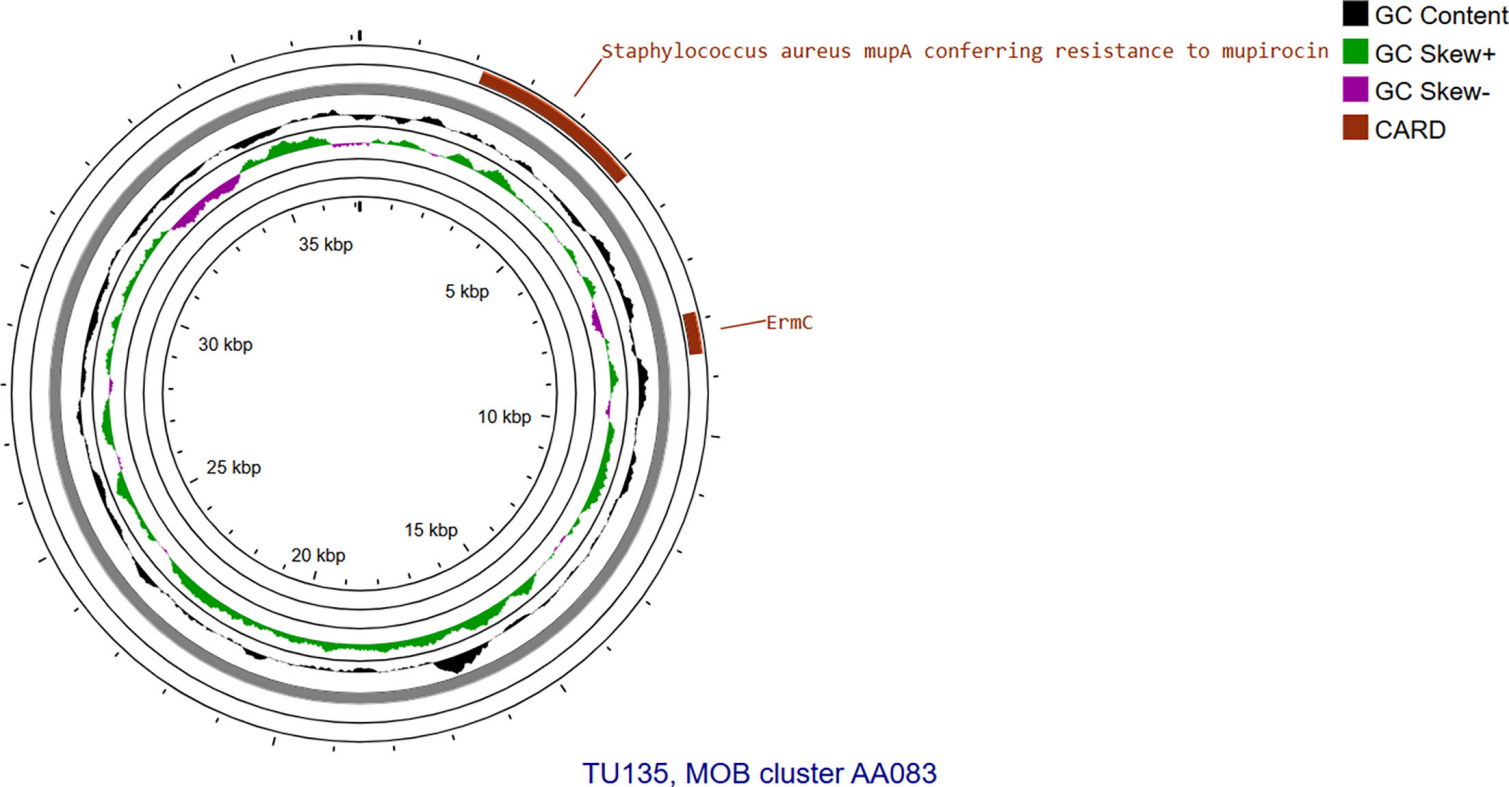
Circular map of the conjugative plasmid AA083 (37 kbp) identified in an MRSA isolate (TU135) recovered from a urine sample during NAP-AMR. Plasmid reconstruction was carried out using MOB-suite v3.1.9, and functional annotation was performed using the CARD accessed via Proksee (https://proksee.ca/). The plasmid harbours two ARGs: *mup*A, associated with mupirocin resistance, and *erm*(C), conferring resistance to macrolide-lincosamide-streptogramin B (MLS-B) antibiotics. MOB-suite mobility analysis classified the plasmid as conjugative, consistent with the presence of both a relaxase and a complete mating pair formation system enabling autonomous horizontal transfer between bacterial cells.

Using MOB-suite, isolates TU135 and TB032 harboured conjugative plasmids which were assigned to MOB cluster AA083 and carried both *erm*(C) and *mup*A genes (Fig. 3). A larger group of isolates (TP018, TU075, TP044-B, TP058-B, TP069-B, TB045-B, TB081-B and TU141-B) harboured non-mobilizable plasmids clustered within AC627 and carried *erm*(C) gene. In addition, isolate TU016 carried *tet*(K) on a mobilizable plasmid clustered within AC333. None of the identified plasmid replicons were found to harbour VF genes. Isolates TU020, TP006-B and TP062-B did not meet the Mash neighbour distance threshold (<0.01) and were not included for further plasmid-related analyses (Table 4).

**Table 4.** Carriage of AMR genes within plasmid clusters and their mobility among MRSA isolates

Isolate	MOB cluster	AMR gene	Mobility
TU016	AC333	*tet*(K)	Mobilizable
TU020	–	–	–
TU135	AA083	*erm*(C), *mup*A	Conjugative
TB032	AA083	*erm*(C), *mup*A	Conjugative
TP018	AC627	*erm*(C)	Non-mobilizable
TU075	AC627	*erm*(C)	Non-mobilizable
TP006-B	–	–	–
TP044-B	AC627	*erm*(C)	Non-mobilizable
TP062-B	–	–	–
TP058-B	AC627	*erm*(C)	Non-mobilizable
TP069-B	AC627	*erm*(C)	Non-mobilizable
TB045-B	AC627	*erm*(C)	Non-mobilizable
TB081-B	AC627	*erm*(C)	Non-mobilizable
TU141-B	AC627	*erm*(C)	Non-mobilizable

MOB cluster refers to plasmid classification based on relaxase gene sequence similarity, reflecting related plasmid mobilization mechanisms. Mobility was categorized as conjugative (encoding complete self-transfer machinery), mobilizable (requiring assistance from conjugative plasmids for transfer) or non-mobilizable (lacking genes necessary for plasmid transfer). The isolate IDs comprise a ‘T’ denoting the country of origin (Tanzania), a letter indicating the specimen type: U (urine), B (blood) and P (pus), and a numeric identifier. A ‘B’ suffix indicates sample collection after NAP-AMR.

### Pangenome analysis of MRSA isolated from clinical samples during and after NAP-AMR

**Fig. 4.**
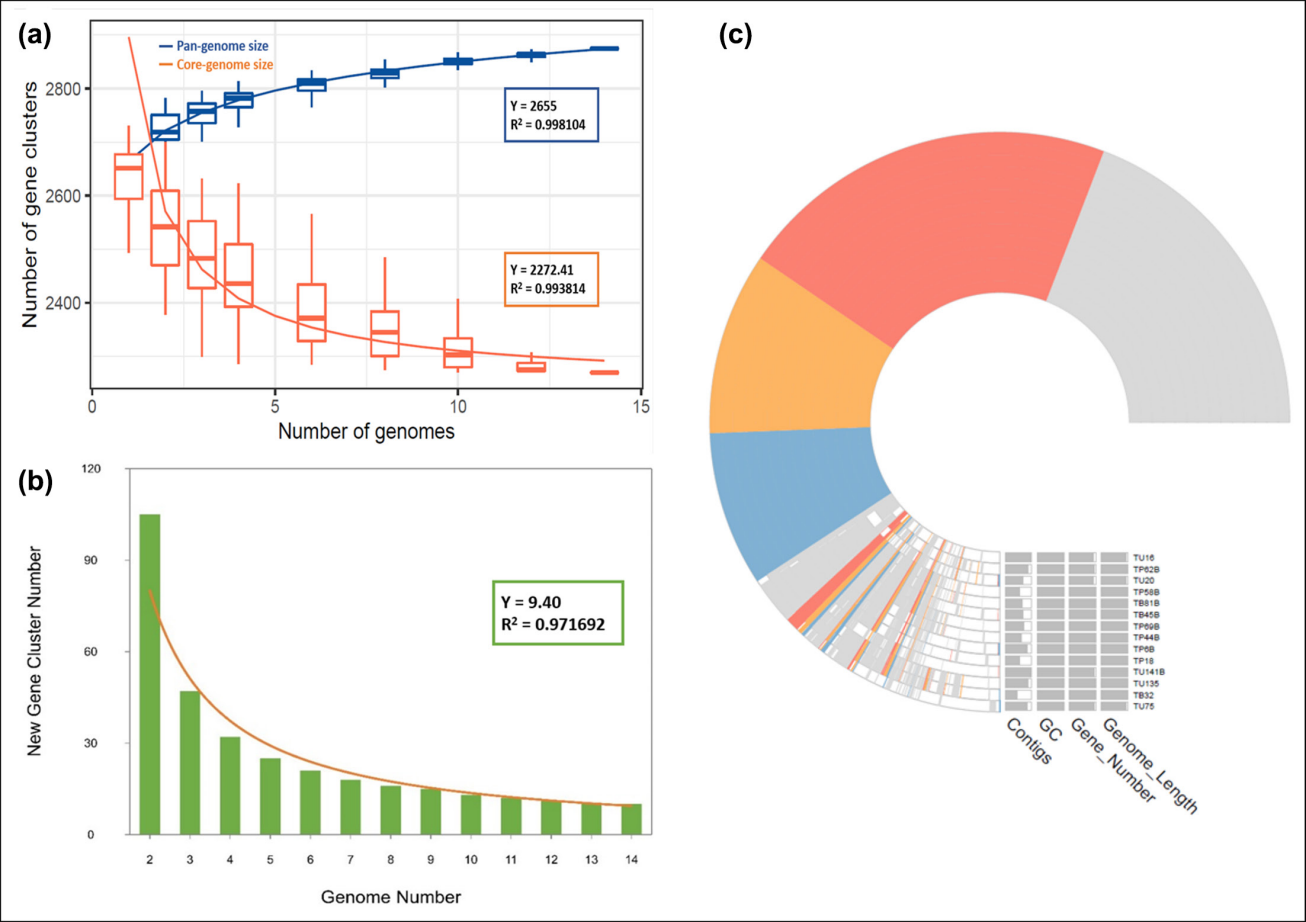
Pangenome and functional annotation of MRSA. (a) Gene accumulation curves of the pangenome (blue) and core genome (orange). Blue and orange boxes denote the MRSA pangenome and core size of each genomic comparison, respectively. (b) Bar graphs showing the number of new genes associated with an increase in the number of MRSA genomes. (c) Comparative genomic analysis of the 14 MRSA isolates. The layers represent individual genomes organized by their phylogenomic relationships. The solid colour bands (red, orange and blue) represent the core genome, whereas the fragmented or faded (grey) segments show the presence or absence of acquired new genes.

A total of 2,989 genes were identified, distributed to 2,258 core genes, 731 accessory genes and another 455 novel genes (Fig. 4). The genes were generally grouped into three functional categories namely metabolisms (carbohydrate, amino acids and lipids metabolism), cellular processing and signalling (cell motility and secretion systems) and information storage and processing (translation, replication and repair). However, on average, each MRSA strain had 2,647 genes in the pan-genome with a minimum of 2,501 genes in the UTI isolate TU20 and a maximum of 2,756 genes in the BSI isolate TB45B.

### Maximum likelihood tree of genome sequences from clinical MRSA isolated during and after NAP-AMR, and from previous studies

**Fig. 5.**
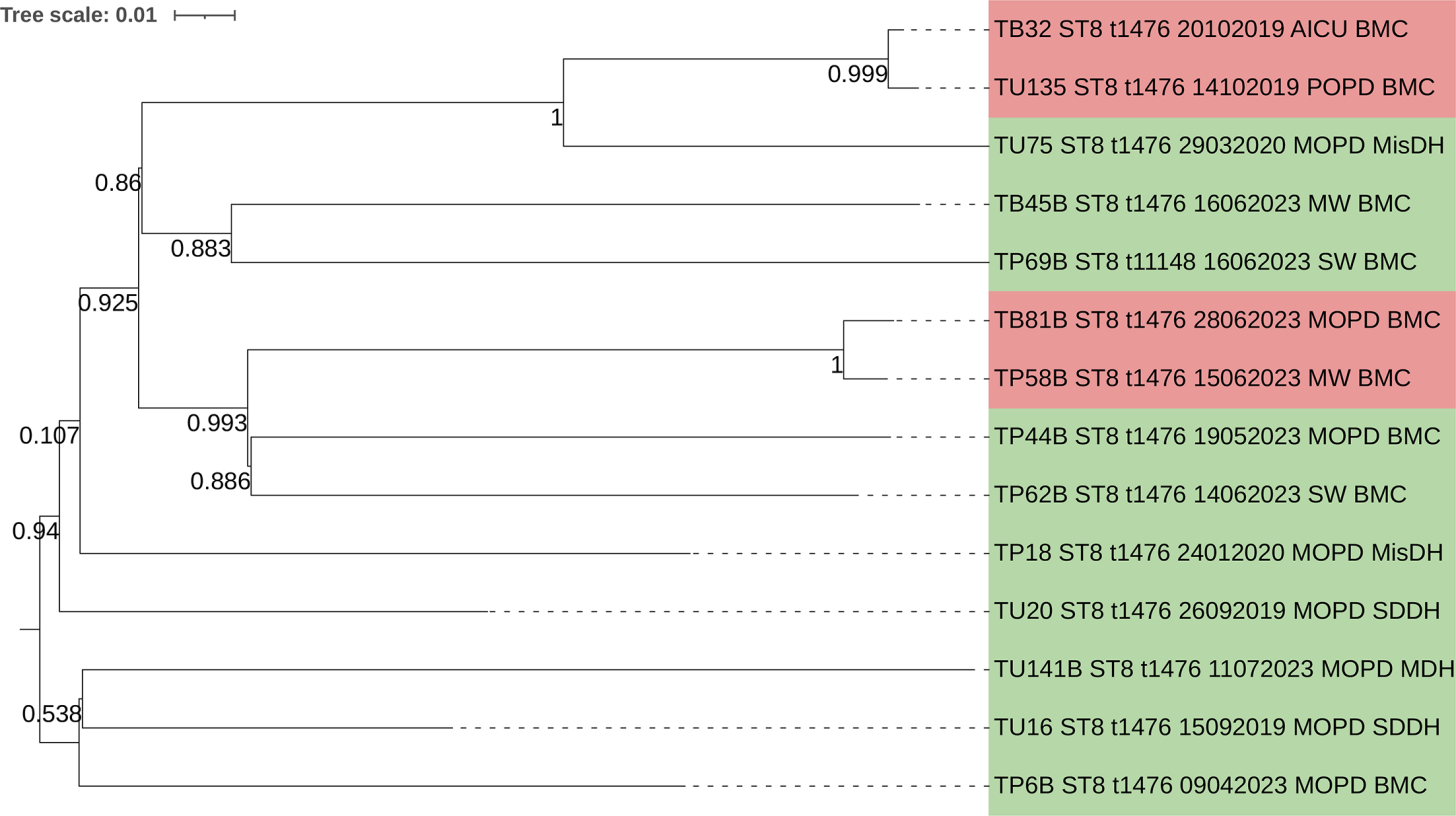
Mid-point rooted SNP-based maximum likelihood phylogenetic tree of MRSA ST8 strains isolated from clinical specimens collected during and after NAP-AMR. The tree was constructed using IQ-TREE v3.1.1, supported by bootstrap values calculated from 1,000 replicates. The scale bar represents 0.01 nucleotide substitutions per site. The isolate IDs comprise a ‘T’ denoting the country of origin (Tanzania), a letter indicating the specimen type: U (urine), B (blood) and P (pus), and a numeric identifier. A ‘B’ suffix indicates sample collection after NAP-AMR. Each genome label, furthermore, includes the ST, spa type, date of isolation (DDMMYYYY), ward or clinic and healthcare facility. Abbreviations: MDH, Magu District Hospital; MisDH, Misungwi District Hospital; MOPD, Medical Outpatient Department; MW, Medical Ward; POPD, Paediatric Outpatient Department; SDDH, Sumve Designated District Hospital; SW, Surgical Ward.

**Fig. 6.**
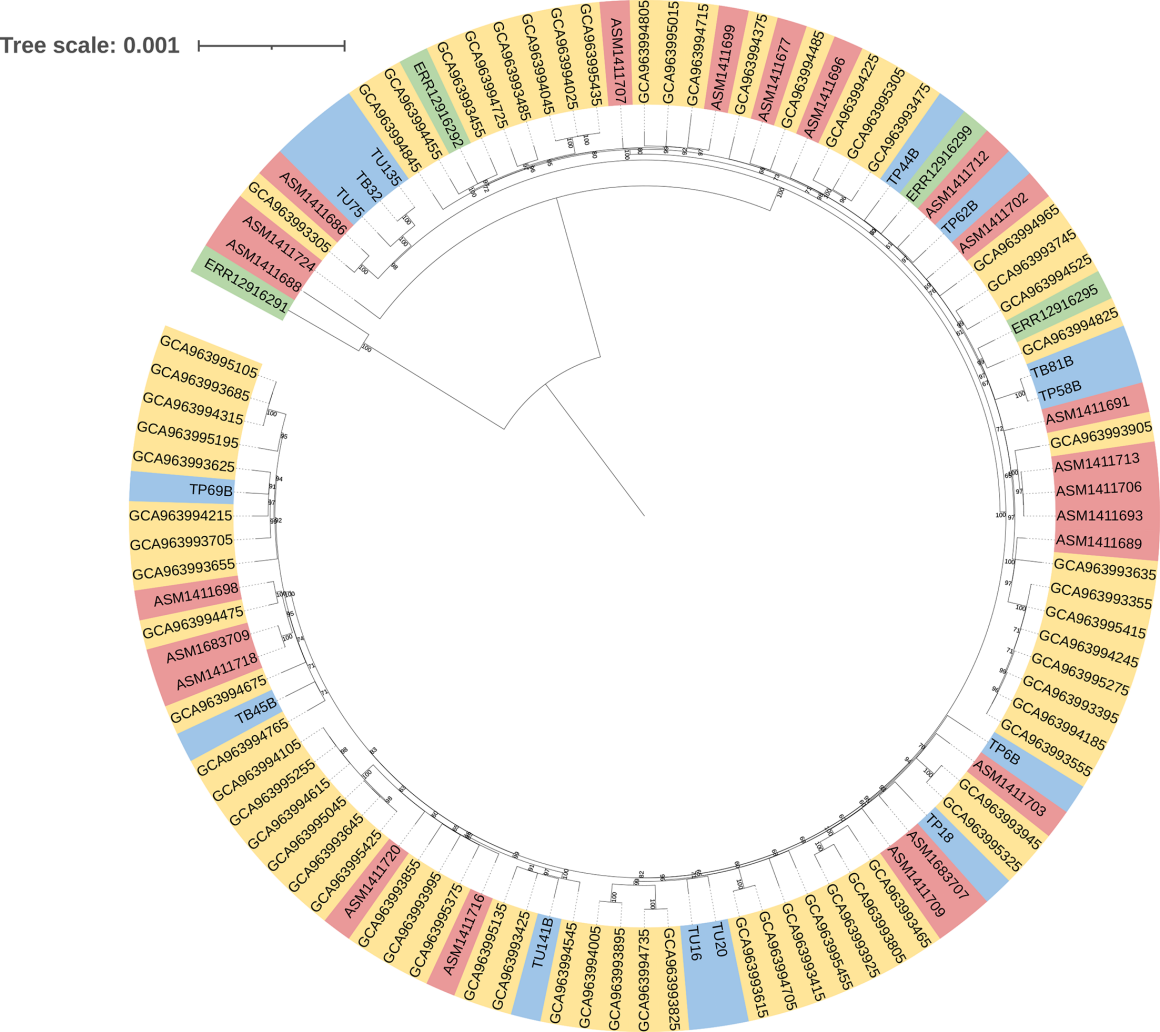
Comparative mid-point rooted SNP-based maximum likelihood phylogenetic tree of MRSA ST8 strains from the present study and previously published studies from Tanzania. The tree was constructed using a maximum likelihood approach and is supported by bootstrap values calculated from 1,000 replicates. The scale bar represents 0.001 nucleotide substitutions per site. Colour coding: light yellow denotes clinical isolates [17]; light red denotes nasal colonization isolates [16]; light green represents clinical isolates [18] from previous published studies in Tanzania and light blue corresponds to isolates from the current study.

SNP differences between the 14 MRSA genomes ranged from a minimum of 0 to a maximum of 99. The maximum likelihood tree revealed two closely related MRSA pairs from BMC, a zonal referral hospital. The first pair consisted of two MRSA isolates with two SNP differences from two patients, which were isolated after NAP-AMR, one from a pus sample (TP58B; 15 June 2023; medical ward) and the other from a blood sample (TB81B; 28 June 2023; medical ward). In contrast, the second pair consisted of two MRSA isolates with 0 SNP difference from two patients, which were isolated during NAP-AMR, one from a urine sample (TU135; 14 October 2019; paediatric ward) and the other from a blood sample [TB32; 20 October 2019; adult Intensive Care Unit (AICU)] (Fig. 5 and Supplementary 1). Additionally, a comparative maximum likelihood phylogenetic analysis and core genome SNP comparisons showed that the MRSA ST8 isolates from this study and those previously reported in Tanzania demonstrated substantial genetic diversity, with ≥30 SNP differences (Fig. 6 and Supplementary 1).

## Discussion

Our small collection of clinical MRSA isolates in Mwanza was dominated by ST8 spa type *t*1476, consistent with previous Tanzanian studies reporting widespread dissemination of this lineage [5,16,18]. This MRSA lineage is well known for its highly virulent and multidrug-resistance traits [42]. While historically linked to community-acquired infections, its strong association with hospital-acquired cases highlights its dual threat [3]. Predominant recovery from pus samples, in line with a previous study [5], reinforces its significant role in SSTIs and its ongoing public health concern.

Our findings, which show the predominance of MRSA ST8 spa type *t*1476, align with a previous study that identified ST8 in 84.0% of sequenced clinical MRSA isolates with co-resistance to quinolones collected across six regional referral hospitals in Tanzania (excluding Mwanza), with the majority being typed as spa type *t*1476 [5]. Another study reported that all 22 sequenced MRSA isolates colonizing HIV-infected adults in Dar es Salaam between April 2017 and May 2018 belonged to ST8, with 95.0% identified as spa type *t*1476 [16]. Additionally, another study from Kilimanjaro reported that 44.4% of sequenced *S. aureus* strains isolated from chronic ulcers between August 2022 and April 2023 belonged to ST8 [18]. Taken together, these findings suggest that MRSA ST8 is widely disseminated in Tanzania, indicating potential expansion and its role in both hospital-acquired and community-acquired MRSA infections, likely linked to individual colonization. In contrast to our findings, the predominant spa types reported in Uganda were *t*645 and *t*4353 with no ST reported [43], whereas in Kenya, ST239 and ST241 (*t*037; both), ST22 (*t*223) and ST8 (*t*064) were most common [44]. Variations in the predominant STs and *spa* types between Tanzania, Uganda and Kenya may stem from differences in environmental conditions and antimicrobial pressure, both of which drive genetic drift and evolutionary changes.

The detection of 110 VF genes across the 14 MRSA isolates underscores the pathogenic potential of these strains. Universally detected virulence genes, such as *aur*, *hlg*A/B/C, *luk*C/D/E and *spl*A/B/E, encode key virulence determinants, including aureolysin, haemolysins, leucocidins and proteases, which are known to contribute to MRSA’s ability to evade host immune responses and cause tissue damage [4,45]. The detection of the *tst* gene in a single MRSA strain (TU75) isolated on 29 March 2020, from a urine sample of an outpatient undergoing ciprofloxacin treatment at the time of sampling is particularly concerning. This gene encodes TSST-1, a super-antigen linked to severe clinical outcomes [4].

The identification of conjugative plasmids (AA083) in isolates TU135 and TB032, carrying both *erm*(C) and *mup*A, highlights their potential role in the horizontal dissemination of AMR genes [46]. In contrast, the predominance of non-mobilizable plasmids within MOB cluster AC627, all harboured *erm*(C) only, suggests the presence of a conserved plasmid backbone with limited intrinsic transfer capability, although their distribution across multiple isolates may also reflect clonal expansion [47]. The detection of a mobilizable plasmid (AC333) carrying *tet*(K) in TU016 further underscores the diversity of plasmid mobility mechanisms within the dataset. Notably, the absence of VF genes on all identified plasmid replicons suggests that VF determinants are likely chromosomally encoded or associated with other mobile genetic elements including transposons or insertion sequences, whereas plasmids primarily contribute to AMR dissemination [48].

The AST results reveal an alarmingly high resistance of MRSA isolates towards widely used antibiotics. For instance, 85.7–100% of the isolates were resistant towards trimethoprim-sulfamethoxazole, levofloxacin, erythromycin and gentamicin, indicating the ineffectiveness of these frontline treatments [49]. Notably, 85.7% of MRSA isolates exhibited iCLI-R, a common co-phenotype in MRSA [50], further complicating the management of MRSA infections. Interestingly, we observed low resistance of MRSA towards mupirocin and fosfomycin, hence a need to reconsider these agents for nasal MRSA decolonization and treatment of uropathogenic MRSA infections among vulnerable patients, respectively [51]. Additionally, no resistance against vancomycin, tigecycline, linezolid, teicoplanin, fusidic acid and daptomycin was observed; hence, these antibiotic agents should be reserved for severe and invasive MRSA infections.

The phenotypic AMR patterns correlated with the genotypic resistance predictions, except for tetracycline. Five out of six tetracycline-resistant strains carried *tet*(K), a well-established tetracycline efflux pump gene, whereas one harboured *mep*A, which encodes an efflux mechanism for fluoroquinolones, tigecyclines and tetracyclines but has a weaker role in tetracycline resistance [52]. The phenotypic susceptibility of other *mep*A-positive strains suggests that *mep*A alone may not confer significant resistance to tetracycline or may require additional regulatory factors [52].

The maximum likelihood phylogenetic analysis revealed genetic diversity among the isolates and identified two pairs of closely related MRSA sampled within days at a zonal referral hospital. Notably, the first pair consisted of two MRSA isolates isolated 6 days apart during NAP-AMR, one from a paediatric outpatient and the other from an AICU patient. The second pair comprised two MRSA isolates obtained from two patients in the medical wards, collected 13 days apart after NAP-AMR. The occurrence of closely related MRSA isolates suggests the acquisition from a common environmental source or a transmission event within the study setting; however, direct person-to-person transmission was not confirmed. Inadequate IPC practices, patient movements and ward transfers, as well as colonization among patients or healthcare workers, may have contributed to the observation; however, these factors were not specifically investigated. The predominance of ST8, a well-known epidemic lineage of *S. aureus*, is linked to its highly virulent and transmissible traits [42], facilitated by a remarkable ability to colonize human hosts [16]. Moreover, its capacity to adapt to hospital environments supports its role in both community- and healthcare-associated infections [42]. This was also supported by pangenome analysis, which revealed substantial genetic diversity among MRSA isolates, driven by a large accessory and novel gene pool. This diversity may contribute to the strains’ ecological versatility, enabling persistence in both healthcare and community settings.

Comparative genomic analysis of MRSA ST8 isolates from this study alongside previously reported Tanzanian MRSA ST8 isolates indicated that they represent separate lineages within ST8. Collectively, these findings suggest ongoing diversification of MRSA ST8 within Tanzania and point to broader regional dissemination [16,18], reinforcing the importance of sustained genomic surveillance to detect potential emerging clones and track MRSA transmission pathways within and beyond healthcare facilities.

## Study limitations

Although the small number of clinical MRSA isolates in the current study may limit the generalizability of these findings, the study provides important insight by revealing the previously uncharacterized local molecular epidemiology of MRSA in a resource-limited setting.

## Conclusion

The predominance of MRSA ST8 spa type *t*1476 underscores stable genetic evolution and transmission in both hospital and community settings. Phylogenetic analysis identified two pairs of closely related ST8 isolates, sampled within days at the same hospital. Therefore, we advocate for sustaining genomic AMR surveillance initiatives in all hospital tiers to foster targeted interventions, such as robust IPC practices, along with effective antimicrobial stewardship programmes, to curb the spread of MRSA isolates.

## Supplementary material

10.1099/acmi.0.001127.v5 Supplementary Material 1.

## Abbreviations

AICU: adult Intensive Care Unit
AMR: antimicrobial resistance
ARG: antimicrobial resistance gene
AST: antimicrobial susceptibility testing
BIC: Bayesian information criteria
BMC: Bugando Medical Centre
BSI: bloodstream infection
CARD: Comprehensive Antibiotic Resistance Database
CUHAS: Catholic University of Health and Allied Sciences
GFF: genetic feature file
iCLI-R: inducible clindamycin resistance
IPC: infection prevention and control
IPGA: integrated prokaryotes genome and pan-genome analysis
MLS-B: macrolide-lincosamide-streptogramin B
MRSA: methicillin-resistant *Staphylococcus aureus*
NAP-AMR: National Action Plan on Antimicrobial Resistance
SSTI: skin and soft tissue infection
TSST-1: toxic shock syndrome toxin-1
UTI: urinary tract infection
VR: virulence factor
WGS: whole-genome sequencing
